# Survey: smartphone-based assessment of cardiovascular diseases using ECG and PPG analysis

**DOI:** 10.1186/s12911-020-01199-7

**Published:** 2020-07-29

**Authors:** Muhammad Shabaan, Kaleem Arshad, Muhammad Yaqub, Fung Jinchao, M. Sultan Zia, Girridhar Reddy Boja, Muazzam Iftikhar, Usman Ghani, Loknath Sai Ambati, Rizwan Munir

**Affiliations:** 1 The University of Gujrat, Gujrat, Pakistan; 2 Beijing University of Technology, Chaoyang District, Beijing, China; 3 Beijing Laboratory of Advanced Information Networks, Beijing, China; 4 The University of Lahore, Gujarat Campus, Gujarat, Pakistan; 5 Dakota State University, Madison, SD USA; 6 Punjab Education Department, Gugarat, Pakistan; 7 Beijing University of Post and Telecommunication, Beijing, China

**Keywords:** Healthcare, Smartphone, electrocardiogram (ECG), Photoplethysmography (PPG), Heart diseases detection

## Abstract

A number of resources, every year, being spent to tackle early detection of cardiac abnormalities which is one of the leading causes of deaths all over the Globe. The challenges for healthcare systems includes early detection, portability and mobility of patients. This paper presents a categorical review of smartphone-based systems that can detect cardiac abnormalities by the analysis of Electrocardiogram (ECG) and Photoplethysmography (PPG) and the limitation and challenges of these system. The ECG based systems can monitor, record and forward signals for analysis and an alarm can be triggered in case of abnormality, however the limitation of smart phone’s processing capabilities, lack of storage and speed of network are major challenges. The systems based on PPG signals are non-invasive and provides mobility and portability. This study aims to critically review the existing systems, their limitation, challenges and possible improvements to serve as a reference for researchers and developers.

## Introduction

Cardiovascular diseases (CVDs) and other heart abnormalities are some of the leading causes of death around the globe. These cardiac abnormalities caused 29.6% of total deaths worldwide in 2010 [1]. The report of the American Heart Association states that almost 17 million people die every year due to cardiac abnormalities, and this number will grow up to 23 million by 2030 [2]. The developing countries are affected particularly, and seven out of every ten deaths will be caused by heart-related abnormalities [3]. Another report claims that heart diseases are the number one cause of deaths by consuming 20 million lives annually. These heart diseases are affecting both developed and developing countries, especially in the urban lifestyle. In the modern era, busy routines, stress at workplaces, an unhealthy diet, smoking, alcohol, and negligence of daily health tips, such as exercise and a healthy diet, are widely adopted. This negligence has shown catastrophic results such as several people even in younger ages (under 35) now having serious heart diseases such as Blood Pressure (BP) disorder, hypertension, cardiac arrest and heart attack [4]. Cardiac arrhythmia is a disorder and abnormal heart rhythm, which leads to a group of heart diseases. To minimize the causalities, the early detection of these cardiac abnormalities is essential. The Golden standard to record and measure the electrical activities of the heart is Electrocardiogram (ECG). Detecting and classifying noises automatically make ECG more robust to use [5]. The ECG is the most widely adopted, economic, and accurate test to measure the performance of the heart. Further, the analysis of ECG is the most effective way for the projection of cardiac abnormalities [4]. There are a few problems with the traditional ECG machines. These are the only hospital or clinic-based machines because of the bulky size, which cannot be moved around easily. It requires a medical professional to conduct the test. These tests record ECG for only 1–2 min, which is not suitable for abnormalities detection because sometimes the arrhythmias are not present at the time of recording. That is why a considerable amount of money is being spent every year on the detection of heart diseases. The report of the American Heart Association [6] claims that the USA alone bears 393.5 billion dollars to address heart diseases, and almost 4 billion dollars of them are spent in hospital emergency sectors in the assessment of non-cardiac causes. The hospitals and healthcare facilities are very far away in developing countries, so people have to travel a lot for the analysis of cardiac functionality. Further, the aged and older adults may not feel it convenient to visit the hospital frequently. The method uses the genetic algorithm process to generate a secret key and to select the best matrix mixing values. This key will be shared between the sender and the receiving message only to ensure that data sources are kept secure and difficult to access by third parties [7]. Most of the heart diseases occur and patient reserves only a few minutes for proper treatment, so the early or in-time detection of these heart diseases is utmost necessary. All these discussed problems lead and enforce researchers to propose new solutions for healthcare monitoring, which can address these issues. A system with traditional key creation techniques such as using a response switch register is protected from a cypher attack that renders it inactive and insecure [8]. It is necessary to implement new low-cost and home-based systems that can continuously monitor cardiac functionalities. Awareness of information can play a major role in addressing cyber attacks by attackers [9]. Thanks to the enhanced Personal Digital Assistants (PDAs) and smartphones, they made it possible. Smartphones nowadays are equipped with enhanced tiny but very powerful sensors, high-speed processors, wireless transmission capabilities, extended storage options, and longlasting batteries. Smartphones are used in business, education, entertainment, social connectivity, and every other field of life. The Smartphone Society of UK states that 93% of their population use smartphones [10].

Online resources [11] reveals that there were 2.1 billion smartphone users in 2016, 2.32 billion in 2017, which will grow to 2.53 billion in 2018, and 2.87 billion by 2020.It attempts to search the capabilities of a comment tool to guess the missing values of various data called random forest and least squares (DRFLLS) [12]. The smartphone users in China and the USA in 2017 were 563 and 223 million, respectively. By 2018, 36% of the total population of the world is projected to use smartphones. Considering this extensive usage and enhanced capabilities of smartphones, researchers proposed and implemented smartphone-based healthcare systems. The purpose of this paper is to review those cardiac monitoring systems which use smartphone directly or indirectly for the analysis of Electrocardiogram (ECG) and Photoplethysmography (PPG). PPG can be defined as the measurement of changes in blood volume [13]. It can be used to measure many heart-related parameters such as Blood Pressure (BP), Heart Rate (HR) and Heart Rate Rhythm, etc. PPG is used in many cardiac related commercial devices such as Pulse oximeters and beat-to-beat BP measurement systems. These smartphones based systems provide mobile and ambulatory monitoring, real-time feedback, and portability, ease of usage, and wireless transmission of data. They can be invasive or noninvasive. The use of built-in or wearable sensors made it possible to acquire ECG or PPG signals. These signals are transmitted using wireless data transmission technologies and processed using various techniques and algorithms. With the technological advancements like computer vision, it is even more evident these day to solve this burning issue. Automatic medical diagnosis is an emerging field of study in the field of computer vision as it provides in-depth analysis on patient’s status [14].

We have divided this review into two significant parts where the first one describes the smartphone-based systems that detect heart diseases and abnormalities by analysis of ECG, whereas the second group measure cardiac parameters and detect abnormalities by the analysis of PPG. The first group is further divided into three subcategories.

1. Analysis of ECG using smartphone

- Recording Visualization systems
- Analysis of ECG on server
- Analysis of ECG on smartphone

2. Analysis of PPG using smartphone

The first subcategory of ECG based systems consist of those early and lately systems that focus on the recording of ECG signals using built-in sensors of a smartphone or other wearable sensors. After the recording, data is sent to the smartphone using wireless transmission technologies such as infrared, Bluetooth, or other radio transmission techniques. These digitized ECG signals are then visualized on PDAs or smartphone screens for analysis. The patient can view the signals and send them to the medical professional/cardiologist for analysis. Unfortunately, the major drawback of these systems i.e., abnormality detection, is still a manual task. It is a time-consuming procedure that is not tolerable in the case of heart diseases. To make the abnormalities detection automatic, researchers have developed systems that fall in the second subcategory. These systems are developed as a client-server infrastructure where smartphone acts as a client. It receives signals from sensors and transmits them to a server via Wi-Fi, GSM, 3G, or 4G. These servers are programmed with ECG analysis algorithms which detect an abnormality and send back the report/alert to patient and doctors. Though these systems make the analysis automatic, a significant drawback is the lack of real-time feedback in case of abnormality detection. Additionally, the traffic on the transmission medium, high cost, and optimum performance of the network and server are always necessary for the system to work. These all problems are addressed in final and third subcategory where ECG signals acquisition, processing, abnormality detection, and generation of alert is performed on the smartphone. This type of system provides continuous analysis, in-time detection of abnormalities, real-time feedback in the form of an alert to patient, caretaker, and doctor.

**Fig. 1:**
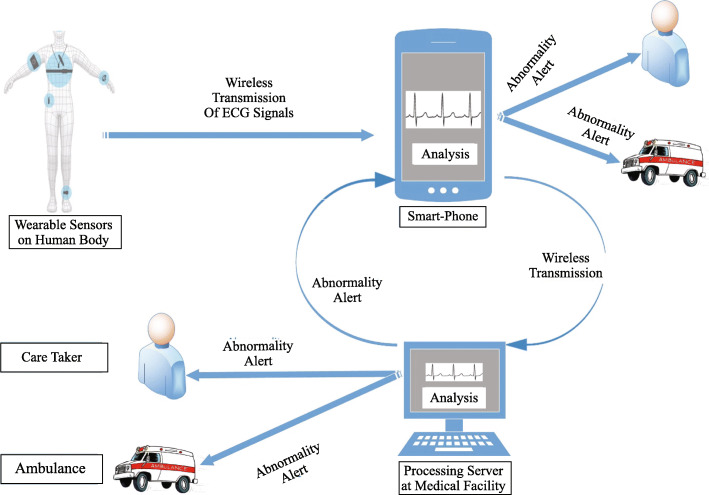
A general representation of smartphone based cardiac monitoring systems

A general representation of these systems is presented in Fig. 1, which consists of all these three types of systems. Section 2 and Section 3 review the smartphone-based systems with analysis of ECG and PPG, respectively. Discussion and open issues are presented in section 4 and section 5, respectively. Finally, the paper is concluded in section 6.

### Electrocardiogram (ECG)

**Fig. 2:**
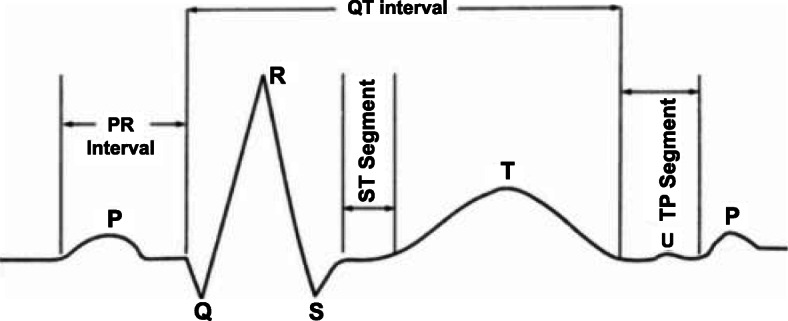
A typical ECG waveform of cardiac cycle

Electrocardiography is the golden standard to record and measure the electrical activities of the heart for a period of using electrodes. The graph of these voltages and time for which they are recorded referred to as Electrocardiogram (ECG). ECG provides a pattern of depolarization and repolarization of heart muscles during each heartbeat [15]. The first ECG test was conducted by Willem Einthoven in 1901 while working in Netherland [16]. Traditionally, ECG machines conduct the test using leads, which are Negative and Positive poles. These leads are attached to the skin surface of the patient’s body and measure electrical activities generated by the human heart. Now a day, ECG is the most widely adopted, economic, and accurate test without any side effects. A typical ECG waveform is presented in Fig. 2. One cardiac cycle in a typical ECG waveform consists of a five-point named P, Q, R, S, and T. Atrial depolarization and P and T waves, respectively represent ventricular repolarization. The collection of three points Q, R and S, named as QRS complex represents ventricular depolarization. The QRS complex is the most important and informative part of the ECG waveform. It is used as a reference point for the detection of other points, and the peak of the R wave is used to estimate heart rate by R-R interval. The upcoming section reviews the works that have been done to digitize this complete procedure of ECG analysis.

#### Recording visualization

**Fig. 3:**
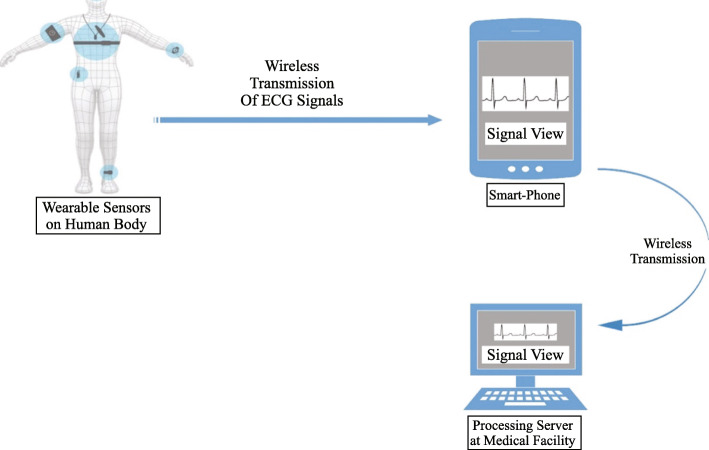
A generic representation of Smartphone based ECG recording visualization systems

Traditionally, cardiologists analyze the graph of ECG and detect abnormality with the reference of amplitudes and frequencies of these waveform points. In this digital era [17], the acquisition, transmission, and analysis of ECG have digitalized. The first attempt to digitalize these systems was the Holter Monitors. They record ECG for 24–48 h, which are stored in a Flash Drive/ memory card for further analysis [18]. They digitalized the procedure but failed to provide real-time monitoring and feedback. Those are bulky in size, expensive in cost, and uncomfortable for patients due to wires, wet electrodes, and high sensitivity to motion artifacts [19]. Zio Patch [20] tried to eliminate Holter’s limitations and monitor the patient’s ECG upto 14 days. The limited signal quality, poor detecting abilities, wet and fixative electrodes are still significant concerns to be addressed. The enhanced features of PDAs and smartphones, with the help of miniaturization, finally made possible the ambulatory monitoring of ECG. The miniaturization is the concept of developing electronic equipment in as smaller and effective size as possible [21]. The recording and visualization of ECG signals on the screen of tablets, PDAs, and smartphones are achieved using robust sensors, wireless transmission technologies such as Bluetooth and advanced GUI. In 2000, Palm Pilot based ECG recorder [22] was introduced, which consists of two modules, namely ECG acquisition and Palm ECG application. Acquisition module record ECG signals using wearable sensors, perform analog to digital conversion, two-stage amplification, and transmit them using the serial port to Palm ECG application. This application, running on PDA, can start, stop, and store ECG recording and patient information. These recorded signals can further be emailed to the doctor for analysis. Microcontrollers also played a critical role in building these systems. A powerful microcontroller (8051) along with ADC0804 data converter, MAX 232 interface chip, and Siemens C55 mobile phone [23] was used to record and visualize ECG signals. The user can start, stop recording and see his recorded signals using automated software on Siemens C55 mobile phone. Usually, wet electrodes are used for ECG recording, which needs a conductive paste to perform the test. They can be allergic, and some patients feel odd to apply them. As a solution, researchers presented a system using fabricated microneedle arrays, dry electrodes, and window smartphone [24]. Though the system was stable, portable, and real-time, the windows mobile phones are not very common in the market. Android operating system of smartphones is the biggest vendor that has 81% of the market [25]. The ECG recording and visual system were presented [26] using a specially developed circuit, and Samsung’s Galaxy Ace, GT –S58308B Android smartphone. The recorded analog ECG signals are converted into digital form and transmitted by the circuit to a smartphone. The Samsung smartphone is used to plot the ECG signals on the screen. Another dry electrodes and the mobile phone-based system was developed [21] using the ECG monitoring circuit and Bluetooth transmission. The electrodes extract weak signals which are then amplified, filtered, digitalized, and transmitted by a specially developed circuit. The waveform of ECG and its points can be viewed distinguishably. The circuits equipped with powerful microcontrollers cannot record signals only but also calculate heart rate [27], using the R-R interval of ECG waveform. This calculated heart rate can be displayed on a screen where signals are plotted and sent to the doctor as a piece of additional information. The quality, flexibility, and ease of ECG acquisition have always been an open issue for researchers. It is necessary to design wearable sensors in such a way that they can quickly be adopted in our daily life routines. By considering the importance of design, a smart shirt [28] was presented, equipped with tiny dry electrodes, chip, flexible antenna, and tiny size battery. These electronics record 12 lead ECG signals, perform digitization, encryption, and transmit them to a smartphone using a low power FSK transmitter. A similar shirt was designed [27] in such a way that equipped electronics can be removed quickly to machine wash the shirt. The electronics of the shirt consist of a circuit board equipped with an ARM microcontroller and Android smartphone application to receive, store, and display ECG signals on the screen. A generic description of the structure of these systems is presented in Fig. 3. All these smartphones based systems fall in the category of telemedicine. Telemedicine is a process of providing interactive medical care, knowledge, and services over a distance through communication technologies [29]. Telemedicine, with the help of these smartphone-based systems, provides continuous monitoring, direct communication between patients and cardiologists, increase efficiency effectiveness, lower costs, and save time for both parties. The concept of telemedicine can play a significant role in developing countries such as India, Pakistan, and Bangladesh. Healthcare facilities and hospitals are very distant in these countries, so that that remote monitoring can facilitate people a lot. A similar system was introduced in Bangladesh [30], where an ECG monitoring system with the help of wearable sensors, an Android smartphone, and the Internet (2G/3G) was developed. This system can transmit ECG signals to the doctor’s computer, where he can analyze them and provide feedback through the Internet. The security, confidentiality, and legal aspects of data, network performance, bandwidth usage, and quality of telecommunication equipment are significant concerns of telemedicine and smartphone-based systems [31], which have to be addressed. These smartphones based cardiac monitoring systems provide patient-doctor interaction [32, 33] with the help of telemedicine to provide real-time feedback. Though these systems digitalized the procedure of cardiac monitoring in a real-time manner, there is no automation in abnormality detection. The cardiologist has to analyze ECG signals manually to detect an abnormality and prescribe precautions and medicines. There is an intense need to automate this detection system. Whenever an abnormal condition occurs, it should be detected by the monitoring system instantly. The upcoming section reviews the systems that have been presented to address this problem.

#### Server based processing of ECG

The era of advanced digital smartphones played a vital role in the development of a complete and automate diagnosis system with the help of seamless integration with low-power, integrated, lightweight, and wearable sensors [34, 35]. This section reviews the systems consisting of client-server architecture where smartphone works at client-side and processing devices (laptops, PC, workstations) at server side. ECG signals acquisition and in-depth analysis for abnormality detection are performed on client-side and server-side, respectively. A real-time ECG monitoring system was presented with GPRS wireless communication technology [10]. The system architecture consists of a client-server model where the client-side has an MCU controller, wireless data transmission module, smartphone, and server-side has a doctor’s workstation. The doctor can view ECG signals and provide real-time feedback. A smartphone and CDMA based [36] system was developed to monitor cardiac health by the analysis of ECG continuously. Wearable sensors generate ECG signals and transmitted to the smartphone. This new system works in one of two ways, at a time. When a patient is in the range of the hospital’s local area network, the WLAN administrator controls the transmission and analysis of ECG by the server. However, when outside the hospital’s WLAN, the smartphone comes into action and controls the transmission of data to the server using TCP/IP protocols. A smartphone-based system eliminates irritating, uncomfortable, and allergic wet electrodes by dry electrodes and provides realtime monitoring [37]. The integrated hardware of this proposed system consists of two active dry ECG electrodes, one RLD electrode, MSP430F5525 microcontroller, micro SD card, Bluetooth module, and HTC G12 mobile phone. The integrated circuit performs data acquisition and transmission to the mobile phone, where the mobile phone is responsible for data forwarding to the server for analysis. Continuous monitoring of data can be very costly, so smartphonebased systems [21, 36] lightly analyze the signals and transmit only when a suspicious condition occurs. The signals generated by sensors or electrodes can be noisy such as motion artifacts, baseline wander, power line interference, and contact noise, which can affect the accurate results of the analysis. This problem was addressed [6] by implementing a preprocessing method in smartphones using a modified Pan-Tompkins QRS complex detection algorithm. The smartphone receives signals, makes them noise-free, and forwards them to the server for in-depth analysis. Whenever an abnormality in ECG is detected, an alarm is generated in the form of SMS or MMS. This alert is the most important part and the main outcome of these systems. The patient, caretakers, and doctors can take immediate action by this alert because, in the case of heart disease, time is the most important factor. The accuracy of abnormality detection procedure is paramount because wrong prediction and treatment can also lead to more serious conditions.

**Fig. 4:**
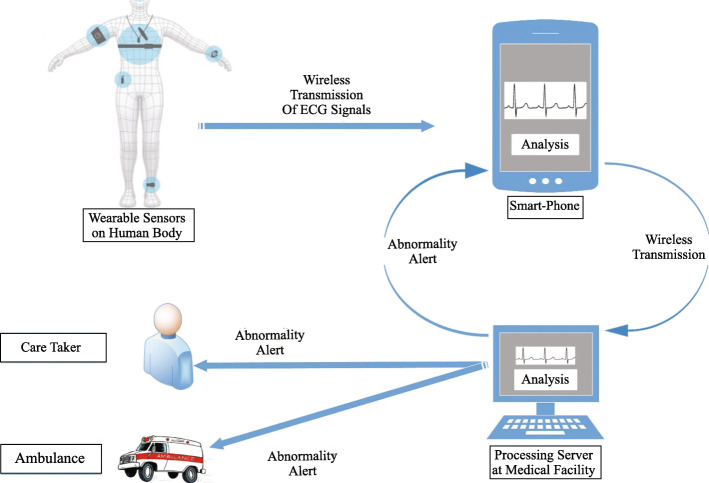
ECG analysis on server for abnormality detection

The smartphone is responsible for receiving signals from wearable sensors and transmit them to a server for analysis. Whenever an abnormality on heart function is detected, sever generates an alert/alarm and sent it back to the smartphone. Some scientific works of this group also implement an alarm mechanism that forward alerts to the caretaker of patient and doctor/ambulance along with the location of the patient. Figure 4 presents the ECG analysis on server for abnormality detection. To make the results more reliable and accurate, researchers implement [25] three different ECG processing algorithms on a mobile phone. The signals generated by sensors are received on the smartphone by Bluetooth and after processing signals are transmitted to the server using 3G, EDGE, or GPRS. Another way to improve accuracy is the adopted selection of processing algorithm. The morphology of the ECG waveform can slightly differ for different patients. A solution was developed where a one-class support vector machine (SVM) was implemented [13] as a local classifier on a smartphone for morphology detection. A two-class SVM was used as a global classifier to detect an abnormality on the server. The critical information of the patient is also stored in PHIMS with an authorized portal for patients and doctors. Another approach was presented where a cardiologist/doctor can select a local and cloud classifier according to the patient’s previous information [32]. Prof. Telman Alive proposed abnormality detection by noise variance, which was implemented [38] using a Windows smartphone. The ECG signals recorded by sensors attached to the body are transmitted to the server (PC) as XLS file. The server performs noise variance and frequency analysis to detect an abnormality. In pregnant women, Fetal Heart Rate (FHR) analysis is very crucial because inaccurate diagnosis is leading to an increased number of cesarean section during labor. A smartphone-based system was presented [39] to monitor pregnant mothers from the analysis abdominal ECG (aECG) using the Pan-Tompkins QRS detection algorithm and Adaptive Nero Fuzzy Interface System (ANFIS). ANFIS is very good for the decision making and verification process, and it can adjust itself to handle noise sources. The MATLAB process the signals in PC and smartphone’s connected session is used to view plots, figures, and results of the analysis. In developing countries, the cost of equipment can also be an issue. Additionally, cardiologists may not be available for many areas. This problem was addressed in Bangladesh, where an ECG processing system was presented based on the simple photograph of the ECG waveform [40]. An individual takes the snapshot of ECG waveform printed on graph paper and transmits it to the processing unit where Discrete Wavelet Transform (DWT), along with many other algorithms process the photograph and detect abnormalities. The abnormality report is provided to the patient on a smartphone using GPRS. The cardiac arrhythmia is the disorder in a normal heartbeat. There are many types of arrhythmia, ranging from simple ones to complex ones. They can lead to many cardiac severe conditions such as heart failure, stroke, heart attack, and finally, death. The in-time detection and treatment of arrhythmias are essential to prevent acute cardiac diseases. The smartphone is playing a vital role in the detection of cardiac arrhythmias. Atrial Fibrillation (AF) is the most common cardiac arrhythmia [41], and reports claim that by 2025, AF will be the reason for 3 million hospitalizations [42]. A smartphonebased AF detection was presented using Wireless Body Sensor Network (WBSN) and Fuzzy Logic [43]. The R-R interval calculates the HR, and a wavelet-based delineation algorithm detects the absence of P wave with a model of the P wave. These two results are then fed to a low complexity classifier Fuzzy logic, which classifies between AF and No-AF signals. Another client-server model based arrhythmia detection system was presented [44], where sensors and smartphones act as client and workstation of the doctor as a server. The smartphone transmits signals to sever where analysis is performed using the Pan-Tompkins algorithm, and the decision tree performs the classification of heartbeats. Myocardial Infarction (MI), also known as a heart attack, is 30% of annual deaths because of heart diseases [45]. The in-time detection of MI is necessary because if it happens, patiently have only a few minutes for the right treatment. Though it is complex to be detected, researchers are also developing smartphone-based systems that use heart rate, heart rate rhythm, heart rate variability, and other arrhythmias detection to talk about MI. Stable, reliable, and in-time detection of Acute Myocardial Infarction (AMI) detection were proposed [46] with the help of portable ECG, cardiac marker analyzer, and Bluetooth enabled smartphone. These devices monitor and analyze the patient’s heart condition continuously, and if any abnormal condition occurs, then data is forwarded to the hospital server. Medical professional staff analyze the data of the client/patient and inform them about necessary actions in case of emergency. Another complete smartphone-based system to detect MI with a newly developed algorithm [47] was presented and validated. The validation was performed on Physikalisch-Technische Bundesanstalt (PTB) diagnostic ECG database using Galaxy Note 2 smartphone and score 97% of sensitivity. MI detection was performed using a smartphone’s built-in accelerometer and gyroscope without any additional hardware [48]. The smartphone was placed on the chest of a patient who lies in a supine position, and analyses were performed SVM and Kernel SVM. These smartphones based systems provide ECG analysis facilities and interaction of patients with medical professionals. An interactive and ambulatory monitoring system was developed [49] where patients and doctors can upload and receive diagnosis reports using point of care. Another real-time ECG processing system provides webbased Graphical User Interface (GUI) not only for patients and doctors but also for caretakers of patients [50]. It enables authorized personnel to monitor the patient’s condition by cardiac parameters such as respiration and heart rate and to facilitate remote diagnosis. The latest system provides real-time ECG monitoring using BodyGateWay (BGW) devices and provides apps for patients and doctors with a cloud environment [33]. Smartphone receives signals from BGW via Bluetooth and transmits them to a cloud server using Wi-Fi. An ID is assigned to every patient, information is encrypted, and in a cloud environment, GUI charts are provided for regular and ventricular events. All these discussed systems provide real-time, ambulatory, and remote monitoring and abnormality detection by the analysis of ECG. The major problem is that feedback of abnormality detection is not realtime because the transmission and processing of data on the server require time. Additionally, the optimal performance of the network and many resources are required for the system to work. If the server or network goes down, the whole system collapse. All these problems are addressed in the upcoming section.

Smartphone Based Processing of ECG This section reviews the systems that continuously monitor cardiac health using a smartphone. These systems can be invasive or non-invasive. The non-invasive diagnosing and follow-up of heart diseases need dependable arterial function measurements from ECG [51]. Biomedical signals are collected using wearable sensors or directly by built-in sensors of the smartphone. The recorded signals are transmitted to a smartphone using wireless transmission technologies such as Bluetooth and Radio frequencies. Visual ECG [33] is a software application that continuously monitors ECG using the Bluetooth module. The inquiry and paging are performed to discover and connect devices and to assign master and slave roles to devices. These signals are processed by algorithms implemented in smartphones in the form of processing apps. A graphical user interface to interact with system and real-time alarm generation systems are provided in the case of abnormality detection [52].

Heart Rate (HR) is an essential and informative cardiac parameter that can early diagnose many upcoming abnormalities [53]. The analysis of the peak of ‘R’ wave and calculation of R-R interval from ECG waveform provides accurate HR and Heart Rate Variability (HRV) analysis. A specially developed hardware (microcontroller, (MCU), AD8232 Heart Monitor, three-lead electrode pads, and HC-06 Bluetooth module) with smartphone App continuously monitor HR and display it on the screen of smartphone [54]. Beat-to-Beat HR was measured by recording the chest vibration of the heart using the accelerometer of a smartphone (Apple, Iphone6) [55]. The Seismcardiogram (SCG) signals were recorded form 9 healthy subjects in two postures: supine and standing. To validate the results, three-lead ECG signals were also simultaneously recorded and gained high than 98% Resting Heart Rate (RHR). A single channel, portable, realtime, low-priced, and low-powered ECG monitoring system was presented, which was integrated with the Arduino microcontroller and connected with a smartphone via Bluetooth [56]. This affordable system provides wireless connectivity up to 9 m and also calculates heart rate from ECG signals. Another device consists of the instrumentation amplifier, bandpass filters, and isolation amplifier, and connected with a smartphone was developed [57] to monitor cardiac health. The ECG signals recorded by electrodes are transmitted to a smartphone for processing and heart rate calculation. The data can be visualized on screen and sent to Google Drive for storage using the 3G mobile network. A classification between six types of beats was performed [58] on the smartphone. The Discrete Wavelet Transform and Higher-Order Statistics were used to extract a set of eleven features from each ECG beats and a Multilayer Perceptron to classify the beats. An individual can be wirelessly monitored within his own home using several sensors to measure different vital signs. A prototype [59] was successfully developed using wearable sensors, smartphones, and algorithms to process data of physiological parameters. The algorithm was tested and found to be accurate and reliable. HeartSense [60] is a ubiquitous system that does not need any specialized hardware to operate. The noise-free heart rate is calculated using a gyroscopic sensor of a smartphone. HeartSense begins with the client holding his cell phone over the chest. The preprocessing module filters the recorded 3-axes signals, and HR is calculated by the heart rate estimation module using a Kalman filter. This HR calculation is the first step to diagnose any arrhythmias. Early detection of arrhythmia is necessary because it can lead to severe several heart abnormalities if left untreated. A smartphone-based system calculates heart rate and additionally classify between Bradycardia (slow HR ¡60 BPM), Tachycardia (fast HR ¿100), and Ventricular Fibrillation [61]. HeartToGo [4] is a smartphone application that receives ECG signals from Alive technology’s state-of-the-art wireless ECG heart monitor. A classification between regular heartbeat and ventricular Fibrillation arrhythmias was performed using the Pan-Tompkins QRS detection algorithm and Artificial Neural Network (ANN). This system can operate in a clinical and non-clinical environment. Continuous monitoring, real-time processing, data compression, long-term storage, and alarm generation using GSM networks were provided as additional features.

**Fig. 5:**
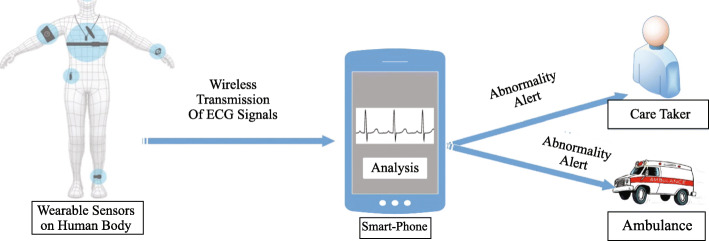
Smartphone Based analysis of ECG

A smartphone-based system integrated with web application process ECG signals using the Pan-Tompkins algorithm on the smartphone. The HR of the patient and other arrhythmias are detected using the R-R interval of the ECG waveform. The mobile application performs the alerting function in case of abnormality detection. It sends messages to the patient, a relative of patient, doctor, nearby hospital within the range of 5 KM and also provides the best route for an ambulance using Google services using mobile cellular network [62]. Figure 5 presents the actual concept of these smartphone-based ECG analysis systems.

Another Hand-Held Device (HHD) based system receives ECG signals from sensors using RF-Radio transmitter and process them. If an abnormal cardiac condition is encountered, the patient will be informed via a clinical alarm station (CAS) [26]. Further, the Mobicare cardio monitoring system is consisting of a wireless ECG sensor, cell phone (Dopod 959 windows) with an embedded real-time ECG analysis algorithm called MobiECG, web-based server, patient’s database and a user interface [15]. MobiECG processes the ECG signals using the QRS complex and QT interval. GUI of the mobile phone provides a graphical representation of QT interval, measured values, detected conditions of Atrial Fibrillation (AF) and Maximum heart rate (MH), and communication portal to provide a doctor to patient communication. An easy to use single lead ECG recording device called “Kardia” was used along the MOBILE-AF trial application to detect AF [63]. The detection of another heart disease called Left Anterior Hemiblock (LAHB), which occurs due to defect left bundle branch, was performed using Bluetooth enabled smartphone and “Amrita Spandanam” [64]. Amrita Spandanam is a wearable low power, low weighted, and three-channel ECG device. The extracted features from the QRS complex are used to build a mathematical model. SVM based classifiers perform the classification between standard and LAHB signals. Instead of traditional complex and complicated algorithms, Heart Attack detection was performed using HR and the sound of the heart [4]. The mobile stethoscope is used to capture the sound of the heart, which should be “Lub Dub” in regular and “murmur” in an abnormal condition. Fuzzy Logic classifies the sounds and displays normal or abnormal status. A patient is continuously being monitored using various parameters such as Blood Pressure (BP), Heart Rate (HR), and weight scale. Whenever an abnormal condition is encountered, a local alarm is generated; if the patient confirms it, an automatic call is dialed to caregivers, doctors, and the nearest ambulance station. GPRS provides the location patient, and accuracy of alarm was ensured by confirmation from the patient [65]. A GUI based monitoring system was developed using Samsung GTN700, where users can select the mood of the system’s operations [64]. The continuously monitoring, history recording, and alarm generation moods can be selected on the ECG signals generated by portable sensors. All these systems calculate HR during rest position, but a measurement during walking and daily life routine can be more helpful. To address this problem, an application for smartphones was developed with a triaxle acceleration sensor and GPS [66]. The system consists of a shared database and a server on the cloud and a smartphone carried by users. Multilayered Neural network is used to process walking data and predict heart rate with machine learning concepts.

Traditionally, ECG sensors are attached to the chest of the subject to record ECG. It is necessary to introduce such design, which contains more likely the accessories we wear in our everyday life such as jewelry and watches. To tackle this problem, a continuous cardiac monitoring system was designed in the design of a necklace connected wirelessly to a smartphone [67]. This system is based on the cardiac vector of Einthoven triangular, which proves that ECG can also be measured from the back of the neck using one lead dry Ag/AgCi electrodes. ECG signals are processed using the Pan-Tompkins algorithm programmed in Android smartphones. Nevertheless, ECG signals are transmitted wirelessly in these systems, which have many issues like integrity, security, and confidentiality. So data was compressed [68] using energy compression with the run-length approach before transmitting to smartphone having. Net framework installed. The range of abnormality alarm is limited while using Bluetooth and GPRS networks. This problem was addressed by developing an algorithm and application-specific integrated circuit (ASIC) [50] for real-time ECG monitoring and Email based alarming approach. All these smartphones based systems provide real-time, ambulatory, home-based, and interactive cardiac monitoring with portability and mobility. The GUI of apps, considerable detection accuracy, and growing processing capabilities and usage of smartphones are primary keys which are improving these systems.

### Photoplethysmography (PPG) analysis using smartphone

Photoplethysmography (PPG) is a non-invasive technique that has many clinical applications such as to measure changes in blood volume, monitoring of Blood Pressure (BP), cardiac output, oxygen saturation, Heart Rate [69], and many other cardiovascular and heart diseases detection [70]. The technique was firstly reported by Hertzman in 1930 [18] by light absorption capability of blood and reflection of signal components. Thanks to the enhanced processing capabilities and built-in cameras and other sensors of smartphones, which made it possible to develop noninvasive cardiac health monitoring systems. Traditionally, ECG has been used for cardiac monitoring, but researchers have proved with experiments that PPG signals have a strong correlation with ECG waveforms [23]. An ECG cycle consists of five points (P, Q, R, S, and T) and three-time intervals (PR, QRS, and QT). These time intervals contain important cardiac information which can be used for initial diagnosis and abnormality detection. The peak to peak interval of PPG is known to be highly correlated with the RR interval of ECG. This section reviews the non-invasive systems which used built-in cameras of the smartphone to record PPG signals and process them in smartphone for cardiac parameters estimation using various processing techniques. The user places his index finger on the built-in standard camera of the smartphone while letting LED flashlight turned on, in most cases. The PPG signals recorded using Motorola Droid Phone [71] by placing the index finger of the subject on camera while letting flashlight turned on and performed recording in 720 × 480 pixel resolution. The green band of the signal was analyzed as well as HRV oxygen saturation and breathing rates were estimated. This system was validated using standard 5-lead electrode configuration, a commercial reflectance pulse oximeter, and a respiration belt. Another green band based analysis was performed using Fast Fourier Transform (FFT). Spectral and Power Spectral Density (PSD) analysis was utilized to calculate HR [72]. The red channel of the PPG signal has more intensity than two others, blue and green. The signals were recorded in 3GP format and then converted into AVI format. Red channel was processed using five algorithms (peak point (PP), valley point (VP), maximum first derivative (M1D), maximum second derivative (M2D), and tangent intersection (TI)) to obtain the pulse-to-pulse interval (PPI) [73]. This system was also validated by ECG acquisition accompanying processing and states that motion artifacts must be eliminated for more accurate results. Another algorithm was developed to measure HR from PPG signals, and an Android smartphone was used to check the robustness, simplicity, and accuracy of the proposed algorithm [74]. Researchers collected 400 recorded samples to test the system, and a medical device called Beurer BC08 was simultaneously used as a reference to validate the accuracy of results. Smartphones now possess high-speed data transmission capabilities and have embedded microprocessors with the capability to connect wirelessly to external devices. Cell phones are widely used in telemonitoring, serving as a conduit for receiving bio-health information [75] from portable medical devices and mobile sensors. The android application was developed, which calculate HR using PPG signals and compare HRs derived from 3 different devices through BlandAltman plots [76]. Also, telemonitoring over distance was also provided for remote monitoring. In-time detection of cardiac arrhythmias is of utmost necessity because it can lead to severe many heart diseases. The PPG signals were recorded by placing an index finger on the camera of the smartphone, and valid HR and morphological trends were extracted during processing. After extracting and analyzing these features, the decision is made up of the K-mean clustering technique between normal, bradycardia, and tachycardia classes [19]. On the detection of any abnormal heart condition, an alarm is generated to stockholders for medical supervision. Another severe cardiac arrhythmia called Atrial Fibrillation (AF) was detected [77] by analyzing HRV and heart rhythm from PPG signals.

Blood Pressure (BP) is another physiological parameter along HRV which can be used to predict cardiac abnormalities. These non-invasive smartphones based systems also work in this field and estimate BP, along with many other physiological parameters. A smartphone-based system was developed to estimate BP from PPG signals using Artificial Neural Network (ANN) [78]. The signals were enhanced using pixel brightness level, and ANN simultaneously extracts systolic BP (SBP) and Diastolic BP (DBP). To achieve easy integration of application and freedom from hardware software architecture, the used ANN is developed in JAVA. Another smartphone-based system performs frequency domain analysis on the PPG signal to calculate HR and extract various informative features. These extracted features from the systolic peak, the valley point, and the Dicrotic notch of the signal are then used to estimate Very low, low, average, high, and very high BP using ANN and SVM. Hypertension detection [79] was performed on the smartphone by analysis of PPG and HR, HRV, and BP estimation. Pan-Tompkins algorithm was used to process the peak of signals and ANN for decision making based on linear and non-linear extracted features. PPG signals can be used to estimate many cardiac and other physiological parameters, but they are easily vulnerable. The low sampling rate of mobile phones, movement of the finger, and variation in pressure on sensor or camera can harm the quality of signals. The baseline drift and other outliers from PPG signals were removed by constructing a mathematical model using a sum of 2 Gaussian functions [80].

PPG signals captured using Nexus 5 Android phones were processed according to this model, and implementation of K-means clustering and ANN proved that it increases the BP estimation accuracy. The smartphone-based systems which record video PPG signals using standard camera can also have very noisy signals. Finite State Machine (FSM) based approach was presented, which estimates the quality of signal before the calculation of heart rate and reject the input if the video segments are too noisy for fair output calculation [20]. Algorithm crop the segments of video and calculate average red pixels and set the threshold to accept or reject the segment using the Mealy Machine, which is a variant of FSM. These PPG based non-invasive systems provide real-time monitoring with portability and mobility. However, the noisy signals due to motion artifacts and accuracy of detection are two major open issues.

## Discussion

The first section of the paper reviews the smartphonebased systems which digitalized the whole procedure. They can record ECG using wearable sensors and display it on the screen of a smartphone or any other PDA. The cardiologist can analyze these signals and diagnose abnormalities. Few of them provide telemedicine facilities for interaction between doctor and patient. The main limitation of these systems is that the diagnosis or abnormality detection process is still manual. What if the cardiologist is not available, then the systems are not effective at all. This problem was addressed by automating the detection of abnormalities using ECG processing algorithms. The signals are transmitted to a server where the abnormality is detected by analysis. Though the procedure is automated, it required much time and the optimal performance of transmission technologies. The time is a more crucial factor in the case of heart diseases, so there should be such a mechanism that alerts the patient in minimum time. So, the systems presented in section 1.3 came into existence and addressed many of these problems. The ECG acquisition, processing, and alert generation are performed locally on the smartphone. The pros and cons of these systems are presented here.

The enhanced sensors, processing capabilities, and wireless transmission of data encourage researchers to develop smartphone-based healthcare systems. The mobility of the patient is a major concern of these days’ healthcare systems, which is achieved by smartphonebased cardiac monitoring systems. These smartphones based system provides ambulatory monitoring and real-time abnormality detection. It is an effective way to minimize causalities by early detection of abnormality. The patient can walk around freely in their homes and workplaces while being monitored continuously. Additionally, alarm/alert systems enable patients, caretakers, and doctors to address the abnormality immediately. The developing countries, which have medical facilities at a very distance, can take more benefit from these systems. People have to travel a lot for a cardiac check-up, which can be eliminated or minimized using smartphone-based monitoring systems. The aged and older people who cannot visit the hospital conveniently can now be monitored from their own homes. Telemedicine can provide healthcare knowledge from a distance using smartphones. A comparative analysis of these three types of ECG analysis systems is presented in Table 1. The scientific and descriptive comparison is performed based on important parameters. These important parameters have an impact on the quality and acceptance of these systems. These parameters are hardware components, wireless communication technology, medical application, portability mobility, Alert/Alarm in case of abnormality detection, interaction b/w doctor and patient, and security of information of the patient. This comparison will provide a scientific review to researchers and developers that what parameters/functionalities of smartphonebased cardiac monitoring systems are still an open issue. The table is categorized into three groups of ECG analysis systems discussed earlier. Two signs “and “indicate presence and absence that characteristic, respectively. A distinction or advantage of these groups is presented in the last column of the table.

**Table 1:**
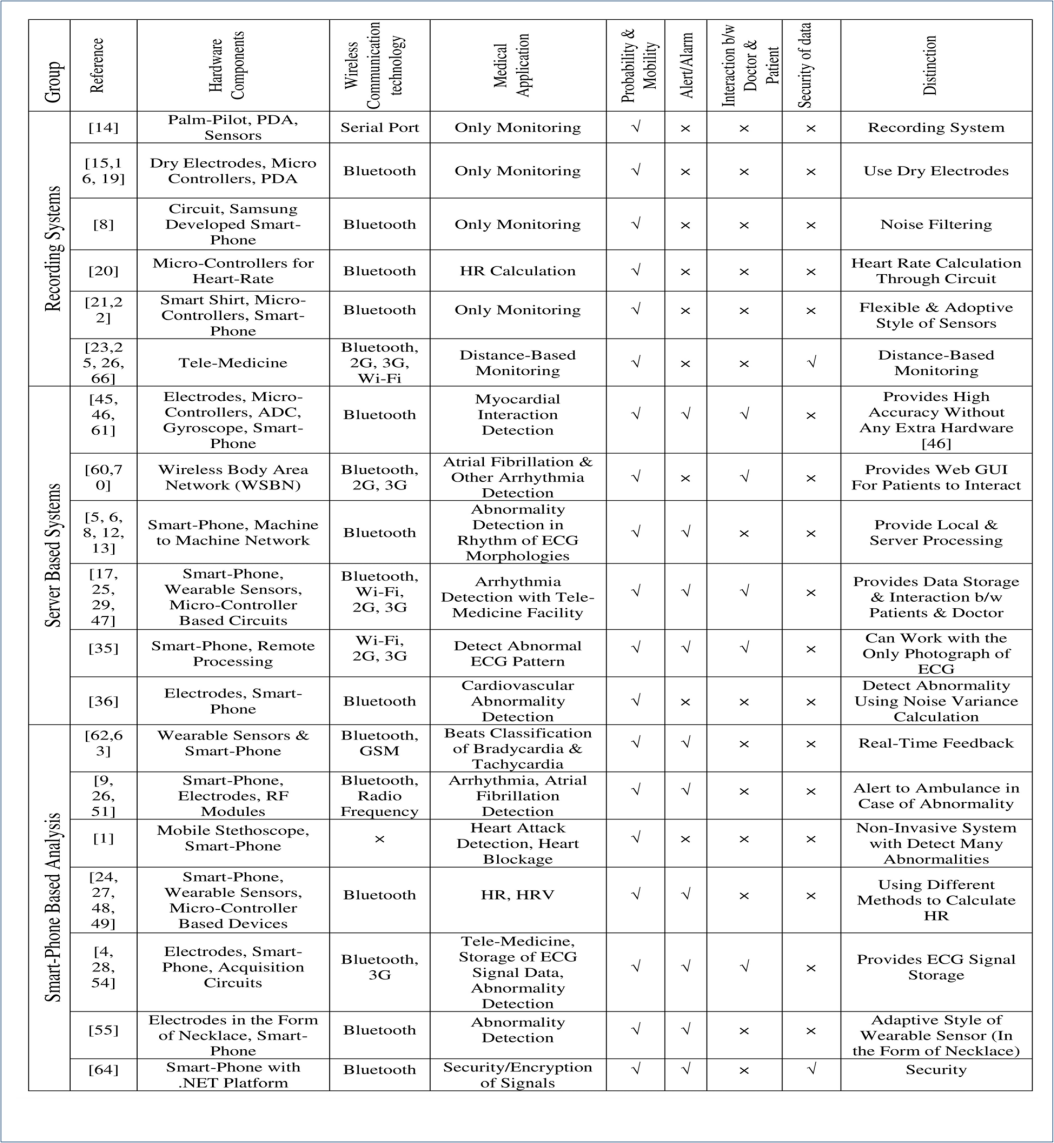
A scientific comparison of smartphone based ECG analysis systems

These systems minimize the resources (money, time) which have been spent on the detection of cardiac abnormalities. Tough the advantages and benefits of these systems are very prominent, but some issues need to be addressed. The design of wearable sensors should be in such a way which can easily be integrated with our daily life routines. People should not feel odd or visually different from others while wearing these sensors. It can be achieved by developing sensors in the design of our dressing accessories. The development of the ECG monitoring device in the form of a necklace [74] and smart shirts [52, 56] were keys to this chain. The range of wireless transmission technologies is another major concern of these systems. Most of them used Bluetooth for signal transmission from wearable sensors to smartphones. Bluetooth has a limited transmission range, which should be interchanged with a high range of wireless transmission technologies to provide more mobility. The security of data during transmission is also an important requirement to be addressed. The information of patients can be confidential, so there should be a mechanism to provide integrity and security of data by encryption techniques. ECG is the most reliable, accurate, and golden standard method to diagnose heart diseases. Though smartphones nowadays have powerful embedded processors, still they have limitations to run ECG processing algorithms. The considerable efforts to develop ECG processing algorithms with low complexity and processing power are the utmost necessity. Most of these systems are limited to HR and BP estimation. There should be an in-depth analysis of ECG to calculate more cardiac parameters and the detection of other heart diseases from ECG features. Wearable sensors and camera of a smartphone during PPG acquisition produce some noises which can affect the accuracy of the analysis. The accurate detection of abnormalities is of utmost necessity because the wrong detection can lead to more complex conditions. So there should be enough mechanism to get rid of noises for accurate processing and reliable results. The alarm/alert mechanism should also be validated, such as confirmed by the patient [70]. A critical analysis of systems has been presented in Table 1. This analysis provides a scientific review to researchers and developers that what parameters/functionalities of smartphone-based cardiac monitoring systems have and these parameters are discussed in detail in this work. PPG analysis based noninvasive systems are also a good source of cardiac parameter detection. They can detect BP, HR, HRV, and other relevant information. Additionally, they do not require any extra hardware and work fine with the built-in camera of smartphones. They provide more portability and mobility. However, they have the primary concern of noises and motion artifacts. Signals get noisy during the acquisition module, and the movement of a finger affects the signal quality and correct analysis [73]. There must be a reasonable mechanism to get rid of these noises to obtain accurate results.

### Open issues

Following are some major issues suggested by reviewing these systems.

1. *Implementation of complex ECG analysis algorithms on smartphones*
  Though smartphones nowadays have improved processors, they are still inadequate for the implementation of complex ECG analysis algorithms. These algorithms used to run in high processors of laptops and PCs. The implementation of these algorithms in a smartphone can improve the analysis, and the number of abnormalities is detected.
2. *Flexible and Adaptive style of wearable sensors*
  The design of wearable sensors should be in such a way which do not look odd and can be used in our daily life routines at homes and workplaces.
3. *Short range of wireless transmission technologies*
  Most of these systems use Bluetooth for wireless transmission of signals from wearable sensors to a smartphone. The limited range of transmission can affect the mobility of patients. Improvement in the range of wireless transmission technologies can increase the mobility of patients.
4. *Accuracy of Diagnosis*
  The accuracy of cardiac parameters and abnormalities detection is still an open issue for researchers. Most of these systems calculate Heart Rate and decide based on it. Other heart diseases which can be detected by the analysis of other points of ECG (P, T) should also be included.

## Conclusion

The enhanced features of the smartphone, such as robust sensors, wireless communication technologies, high-speed processors, and a growing number of usage, made use of smartphones very vital in cardiac health monitoring. A categorical review of smartphone-based systems for real-time cardiac monitoring and abnormality detection is presented in this paper. The primary focal point was the analysis of ECG for heart abnormality detection using wearable sensors and smartphones. Review of ECG based system is divided into three sub-categories named as 1) Recording visualization systems 2) Server-based processing of ECG 3) Smartphone-based processing of ECG. The systems reviewed in the first group can record ECG signals and show them on the screen of a smartphone or any other PDA. The second group review those systems which perform analysis of ECG signals by transmitting them to a processing server at a medical facility. Furthermore, finally, local processing and heart abnormality detection are performed in smartphone-based processing systems, which are reviewed in the third group. Additionally, PPG based non-invasive systems are presented in section two, which can perform real-time monitoring and estimation of cardiac parameters by placing the index finger of the subject on the camera of a smartphone. These systems provide ambulatory, continuous, and real-time heart monitoring and abnormality detection with portability and mobility of patients. Different types of noises and artifact occurred during signal acquisition can affect the accuracy of diagnosis. Telemedicine implemented in these smartphone-based systems provides distance based monitoring and guidelines for patients, especially in less developed rural areas. The short-range of wireless communication technologies such as Bluetooth, implementation of complex ECG analysis algorithms in smartphones, flexible and adaptive design of wearable sensors, fewer abnormalities/disease detection, and security of data are significant issues that need to be addressed. The concept of tele-medicine for distance- based monitoring and alarm generation in case of abnormality detection makes possible the in-time de- tection and treatment of abnormalities to minimize casualties. This study aims to serve as a reference for researchers and developers to provide knowledge of the limitations of existing systems and future im- provements.

## Data Availability

Not applicable.

## References

[1] Cardiovascular diseases. https://www.who.int/health-topics/cardiovascular-diseases/. Accessed on 12 Dec 2019.

[2] Lloyd-Jones D, Adams R, Carnethon M, De Simone G, Ferguson TB, Flegal K, Ford E, Furie K, Go A, Writing Group Members (2009). Heart disease and stroke statistics—2009 update: a report from the american heart association statistics committee and stroke statistics subcommittee. Circulation.

[3] Doka KJ (2014). Living with grief: after sudden loss suicide, homicide, accident, heart attack, stroke.

[4] Ashrafuzzaman M, Huq MM, Chakraborty C, Khan MRM, Tabassum T, Hasan R (2013). Heart attack detection using smart phone. Int J Technol Enhance Emerg Eng Res.

[5] Satija U, Ramkumar B, Manikandan MS (2017). Automated ecg noise detection and classification system for unsupervised healthcare monitoring. IEEE J Biomed Health Inform.

[6] Gakare PK, Patel AM, Vaghela JR, Awale R (2012). Real time feature extraction of ecg signal on android platform. 2012 International Conference on Communication, Information & Computing Technology (ICCICT).

[7] Al-Janabi S, Al-Shourbaji I (2016). A hybrid image steganography method based on genetic algorithm. 2016 7th International Conference On Sciences Of Electronics, Technologies of Information and Telecommunications (SETIT).

[8] Ali SH (2013). Novel approach for generating the key of stream cipher system using random forest data mining algorithm. 2013 Sixth International Conference on Developments in eSystems Engineering.

[9] Al-Janabi S, Al-Shourbaji I (2016). A study of cyber security awareness in educational environment in the middle east. J Inf Knowl Manag.

[10] Zhang K, Song L, Lu D (2011). Design of remote ecg monitoring system based on gprs. Proceedings of 2011 International Conference on Computer Science and Network Technology.

[11] What is tele-medicine? https://www.telemedicine.com/about/what-is-telemedicine/. [Online; Accessed 5 Oct 2013].

[12] Al-Janabi S, Alkaim AF (2020). A nifty collaborative analysis to predicting a novel tool (drflls) for missing values estimation. Soft Comput.

[13] Goh K, Lavanya J, Tan E, Soh C, Kim Y (2006). A pda-based ecg beat detector for home cardiac care. 2005 IEEE Engineering in Medicine and Biology 27th Annual Conference.

[14] Thevenot J, L’opez MB, Hadid A (2017). A survey on computer vision for assistive medical diagnosis from faces. IEEE J Biomed Health Inform.

[15] Chen X, Ho CT, Lim ET, Kyaw T (2007). Cellular phone based online ecg processing for ambulatory and continuous detection. 2007 Computers In cardiology.

[16] Gao H, Duan X, Guo X, Huang A, Jiao B (2013). Design and tests of a smartphones-based multi-lead ecg monitoring system. 2013 35th Annual International Conference of the IEEE Engineering in Medicine and Biology Society (EMBC).

[17] Hong S, Kwon H, Sang HC, Park KS (2018). Intelligent system for drowsiness recognition based on ear canal electroencephalography with photoplethysmography and electrocardiography 453.

[18] Hertzman AB (1937). Observations on the finger volume pulse recorded photoelectrically. Am J Phys.

[19] Ukil A, Bandyopadhyay S, Puri C, Pal A (2016). Heart-trend: an affordable heart condition monitoring system exploiting morphological pattern. 2016 IEEE International Conference on Acoustics, Speech and Signal Processing (ICASSP).

[20] Pal A, Sinha A, Dutta Choudhury A, Chattopadyay T, Visvanathan A (2013). A robust heart rate detection using smart-phone video. Proceedings of the 3rd ACM MobiHoc Workshop on Pervasive Wireless Healthcare.

[21] Kai L, Zhang X, Wang Y, Suibiao H, Ning G, Wangyong P, Bin L, Chen H (2011). A system of portable ecg monitoring based on bluetooth mobile phone. 2011 IEEE International Symposium on IT in Medicine and Education.

[22] Zou Y, Guo Z (2000). A palm pilot based pocket ecg recorder. Proceedings 2000 IEEE EMBS International Conference on Information Technology Applications in Biomedicine. ITAB-ITIS 2000. Joint Meeting Third IEEE EMBS International Conference on Information Technol.

[23] Banerjee R, Sinha A, Choudhury AD, Visvanathan A (2014). Photoecg: photoplethysmographyto estimate ecg parameters. 2014 IEEE International Conference on Acoustics, Speech and Signal Processing (ICASSP).

[24] Engin M, Yamaner Y, Engin EZ (2005). A biotelemetric system for human ecg measurements. Measurement.

[25] Secerbegovic A, Mujˇci’c A., Suljanovi’c N, Nurkic M, Tasic J. The research mhealth platform for ecg monitoring. In: Proceedings of the 11th International Conference on Telecommunications. IEEE; 2011. pp. 103–108.

[26] Fensli R, Gunnarson E, Hejlesen O (2004). A wireless ecg system for continuous event recording and communication to a clinical alarm station. The 26th Annual International Conference of the IEEE Engineering in Medicine and Biology Society.

[27] Gonzales L, Walker K, Keller K, Beckman D, Goodell H, Wright G, Rhone C, Emery A, Gupta R (2015). Textile sensor system for electrocardiogram monitoring. 2015 IEEE Virtual Conference on Applications of Commercial Sensors (VCACS).

[28] Morrison T, Silver J, Otis B (2014). A single-chip encrypted wireless 12-lead ecg smart shirt for continuous health monitoring. 2014 symposium on VLSI circuits digest of technical papers.

[29] Bashshur RL (1995). On the definition and evaluation of telemedicine. Telemed J.

[30] Busra US, Rahman MZ (2014). Mobile phone based telemedicine service for rural Bangladesh: Ecg. 16th Int’l Conf. Computer and Information Technology.

[31] Hu PJ, Chau PY, Sheng ORL, Tam KY (1999). Examining the technology acceptance model using physician acceptance of telemedicine technology. J Manag Inf Syst.

[32] Xie B, Shen L (2014). An wearable ecg analysis system with novel interactive method. 2014 International Conference on Intelligent Environments.

[33] Tsamis G, Grammatikakis MD, Papagrigoriou A, Petrakis P, Piperaki V, Mouzakitis A, Coppola M (2017). Soft real-time smartphone ecg processing. 2017 12th IEEE International Symposium on Industrial Embedded Systems (SIES).

[34] Jayalakshmi R, Mahalingam D, Rajeswari A (2014). Advanced health monitoring and receiving using smartphone in global networks.

[35] Wu W, Pirbhulal S, Zhang H, Mukhopadhyay SC (2018). Quantitative assessment for self-tracking of acute stress based on triangulation principle in a wearable sensor system. IEEE J Biomed Health Inform PP.

[36] Chung W-Y, Yau C-L, Shin K-S, Myllyla R (2007). A cell phone based health monitoring system with self analysis processor using wireless sensor network technology. 2007 29th Annual International Conference of the IEEE Engineering in Medicine and Biology Society.

[37] Xiang Chen X, Lv Y, Fang RRZ, Hong Xia S, Li H, Tian L (2012). A wireless noncontact ecg detection system based on capacitive coupling. 2012 IEEE 14th International Conference on e-Health Networking, Applications and Services (Healthcom).

[38] Aliev T, Babayev T, Sabziev E, Pashayev A, Alizada T (2012). Monitoring of condition of the cardiovascular system by means of mobile phones using ecg noise variance. 2012 IV International Conference “Problems of Cybernetics and Informatics”(PCI).

[39] Marchon N, Naik G (2016). Detection of fetal heart rate using anfis displayed on a smartphone. 2016 IEEE Region 10 Conference (TENCON).

[40] Mitra RN, Pramanik S, Mitra S, Chaudhuri BB (2012). A robust technique for delineation and features extraction of ecg signal from mobile-phone photography. 2012 International Conference on Communications, Devices and Intelligent Systems (CODIS).

[41] Benjamin EJ, Chen P-S, Bild DE, Mascette AM, Albert CM, Alonso A, Calkins H, Connolly SJ, Curtis AB, Darbar D (2009). Prevention of atrial fibrillation: report from a national heart, lung, and blood institute workshop. Circulation.

[42] Wattingney W, Croft J (2003). Atrial fibrillation hospitalizations triple since 1985, will continue to climb.

[43] Rinc’on F, Grassi PR, Khaled N, Atienza D, Sciuto D (2012). Automated real-time atrial fibrillation detection on a wearable wireless sensor platform. 2012 Annual International Conference of the IEEE Engineering in Medicine and Biology Society.

[44] Park J, Lee K, Kang K (2014). Intelligent electrocardiogram monitoring system for early arrhythmia detection. 2014 IEEE 28th International Conference on Advanced Information Networking and Applications.

[45] Ati M (2014). Knowledge capturing in autonomous system design for chronic disease risk assessment. 2014 IEEE Conference on Biomedical Engineering and Sciences (IECBES).

[46] Lee J, Jung J, Lee J, Kim YT (2014). Acute myocardial infarction detection system using ecg signal and cardiac marker detection. SENSORS, 2014 IEEE.

[47] Makki MM, Saade GA, Altouma AG, Al-Terkawi S, Baobeid A, Tafreshi R (2014). Acquiring and analyzing electrocardiograms via smartphone to detect cardiovascular abnormalities. IEEE-EMBS International Conference on Biomedical and Health Informatics (BHI).

[48] Lahdenoja O, Koivisto T, Tadi MJ, Iftikhar Z, Hurnanen T, Vasankari T, Kiviniemi T, Airaksinen J, P¨ank¨a¨al¨a M. A smartphone-only solution for detecting indications of acute myocardial infarction. In: 2017 IEEE EMBS International Conference on Biomedical & Health Informatics (BHI). IEEE; 2017. pp. 197–200.

[49] Bansal A, Kumar S, Bajpai A, Tiwari VN, Nayak M, Venkatesan S, Narayanan R (2015). Remote health monitoring system for detecting cardiac disorders. IET Syst Biol.

[50] Zhang Y, Liu H, Su X, Jiang P, Wei D (2015). Remote mobile health monitoring system based on smart phone and browser/server structure. J Healthcare Eng.

[51] Veye F, Mestre S, Perez-Martin A, Triboulet J (2014). Possibility of non-invasive blood pressure estimation by measurements of force and arteries diameter. IEEE-EMBS International Conference on Biomedical and Health Informatics (BHI).

[52] Oresko JJ, Jin Z, Cheng J, Huang S, Sun Y, Duschl H, Cheng AC (2010). A wearable smartphone-based platform for real-time cardiovascular disease detection via electrocardiogram processing. IEEE Trans Inf Technol Biomed.

[53] Wilkinson IB, MacCallum H, Flint L, Cockcroft JR, Newby DE, Webb DJ (2000). The influence of heart rate on augmentation index and central arterial pressure in humans. J Physiol.

[54] Turner J, Zellner C, Khan T, Yelamarthi K (2017). Continuous heart rate monitoring using smartphone. 2017 IEEE International Conference on Electro Information Technology (EIT).

[55] Landreani F, Mart’ın-Yebra A, Casellato C, Frigo C, Pavan E, Migeotte P-F, Caiani EG (2016). Beat-to-beat heart rate detection by smartphone’s accelerometers: validation with ecg. 2016 38th Annual International Conference of the IEEE Engineering in Medicine and Biology society (EMBC).

[56] Ahamed MA, Hasan MK, Alam MS (2015). Design and implementation of low cost ecg monitoring system for the patient using smartphone. 2015 International Conference on Electrical & Electronic Engineering (ICEEE).

[57] Brucal SGE, Clamor G, Pasiliao L, Soriano J, Varilla L (2016). Portable electrocardiogram device using android smartphone. 2016 38th Annual International Conference of the IEEE Engineering in Medicine and Biology Society (EMBC).

[58] Varella FA, de Lima GL, Iochpe C, Roesler V (2011). A method for the automatic classification of ecg beat on mobile phones. 2011 24th International Symposium on Computer-Based Medical Systems (CBMS).

[59] Malhi K, Mukhopadhyay SC, Schnepper J, Haefke M, Ewald H (2010). A zigbee-based wearable physiological parameters monitoring system. IEEE Sensors J.

[60] Mohamed R, Youssef M (2017). Heartsense: Ubiquitous accurate multi-modal fusion-based heart rate estimation using smartphones. Proc ACM Interact Mobile Wearable Ubiquitous Technol.

[61] Alzate EB, Martinez FM (2010). Ecg monitoring system based on arm9 and mobile phone technologies. 2010 IEEE ANDESCON.

[62] Rodr’ıguez-Gallo U, Charco-Castellanos F, Tovar-Corona B, de la Cruz-Sosa C, Alvarado-Serrano C (2015). Heart rate measurement system using mobile devices to alert arrhythmias. 2015 12th International Conference on Electrical Engineering, Computing Science and Automatic Control (CCE).

[63] Treskes RW, Gielen W, Wermer MJ, Grauss RW, van Alem AP, Dehnavi RA, Kirchhof CJ, van der Velde ET, Maan AC, Wolterbeek R (2017). Mobile phones in cryptogenic stroke patients bringing single lead ecgs for atrial fibrillation detection (mobile-af): study protocol for a randomised controlled trial. Trials.

[64] Arunan A, Pathinarupothi RK, Ramesh MV (2016). A real-time detection and warning of cardiovascular disease lahb for a wearable wireless ecg device. 2016 IEEE-EMBS International Conference on Biomedical and Health Informatics (BHI).

[65] Gay V, Leijdekkers P (2007). A health monitoring system using smart phones and wearable sensors. Int J ARM.

[66] Sumida M, Mizumoto T, Yasumoto K (2013). Estimating heart rate variation during walking with smartphone. Proceedings of the 2013 ACM International Joint Conference on Pervasive and Ubiquitous Computing.

[67] Iskandar AA, Kolla R, Schilling K, Voelker W (2016). A wearable 1-lead necklace ecg for continuous heart rate monitoring. 2016 IEEE 18th International Conference on e-Health Networking, Applications and Services (Healthcom).

[68] Rezazadeh IM, Parvaresh S, Zargar MEH, Proulx J (2011). Ecg data compression for mobile phone tele-cardiology applications using. Net framework. 2011 1st Middle East Conference on Biomedical Engineering.

[69] Youssef A, Pena Fernandez A, Wasserman L, Biernot S, Bleich A, Hartung J, Norton T (2019). Heart rate monitoring in pigs using photo pethysmography (ppg) technology. Precision Livestock Farm.

[70] Uguz DU, Venema B, Leonhardt S, Teichmann D (2019). Multifunctional photoplethysmography sensor design for respiratory and cardiovascular diagnosis.

[71] Scully CG, Lee J, Meyer J, Gorbach AM, Granquist-Fraser D, Mendelson Y, Chon KH (2011). Physiological parameter monitoring from optical recordings with a mobile phone. IEEE Trans Biomed Eng.

[72] Jonathan E, Leahy MJ (2011). Cellular phone-based photoplethysmographic imaging. J Biophotonics.

[73] Pereira T, Gadhoumi K, Ma M, Xiuyun L, Xiao R, Colorado RA, Keenan KJ, Meisel K, Hu X. A supervised approach to robust photoplethysmography quality assessment. IEEE J Biomed Health Inform, 1–1. 10.1109/JBHI.2019.2909065 PMC9553283 30951482

[74] Hoan NV, Park J-H, Lee S-H, Kwon K-R (2017). Real-time hear t rate measur ement based on photoplethysmogr aphy using android smar tphone camera. Multimedia Soc J.

[75] Francesco R, Sabrina C, Alessandro O, Sebastiano B (2018). An advanced bio-inspired photoplethysmography (ppg) and ecg pattern recognition system for medical assessment. Sensors.

[76] Gregoski MJ, Mueller M, Vertegel A, Shaporev A, Jackson BB, Frenzel RM, Sprehn SM, Treiber FA (2012). Development and validation of a smartphone heart rate acquisition application for health promotion and wellness telehealth applications. Int J Telemed Appl.

[77] Lagido RB, Lobo J, Leite S, Sousa C, Ferreira L, Silva-Cardoso J (2014). Using the smartphone camera to monitor heart rate and rhythm in heart failure patients. IEEE-EMBS International Conference on Biomedical and Health Informatics (BHI).

[78] Lamonaca F, Barbe K, Kurylyak Y, Grimaldi D, Van Moer W, Furfaro A, Spagnuolo V (2013). Application of the artificial neural network for blood pressure evaluation with smartphones. 2013 IEEE 7th International Conference on Intelligent Data Acquisition and Advanced Computing Systems (IDAACS).

[79] Gaurav A, Maheedhar M, Tiwari VN, Narayanan R (2016). Cuff-less ppg based continuous blood pressure monitoring—a smartphone based approach. 2016 38th Annual International Conference of the IEEE Engineering in Medicine and Biology Society (EMBC).

[80] Banerjee R, Ghose A, Choudhury AD, Sinha A, Pal A (2015). Noise cleaning and gaussian modeling of smart phone photoplethysmogram to improve blood pressure estimation. 2015 IEEE International Conference on Acoustics, Speech and Signal Processing (ICASSP).

