# Correction to: Survey: smartphone-based assessment of cardiovascular diseases using ECG and PPG analysis

**DOI:** 10.1186/s12911-020-01229-4

**Published:** 2020-09-10

**Authors:** Muhammad Shabaan, Kaleem Arshid, Muhammad Yaqub, Feng Jinchao, M. Sultan Zia, Giridhar Reddy Boja, Muazzam Iftikhar, Usman Ghani, Loknath Sai Ambati, Rizwan Munir

**Affiliations:** 1 The University of Gujrat, Gujrat, Pakistan; 2 Beijing University of Technology, Chaoyang District, Beijing, China; 3 Beijing Laboratory of Advanced Information Networks, Beijing, China; 4 The University of Lahore, Gujarat Campus, Gujarat, Pakistan; 5 Dakota State University, Madison, SD USA; 6 Punjab Education Department, Gugarat, Pakistan; 7 Beijing University of Post and Telecommunication, Beijing, China

**Correction to: BMC Med Inform Decis Mak (2020) 20:177**

**https://doi.org/10.1186/s12911-020-01199-7**

Following publication of the original article [1], the authors identified errors in the names of several authors.

The incorrect names were: Fung Jinchao, Kaleem Arshad, and Girridhar Reddy Boja

The correct author names are: Feng Jinchao, Kaleem Arshid, and Giridhar Reddy Boja

The author group has been updated above and the original article [1] has been corrected.
